# Advances on Implementation Science in Health: Insights on trajectory, lessons learned and opportunities from the Portuguese experience

**DOI:** 10.1590/1518-8345.0000.5054

**Published:** 2026-07-31

**Authors:** Sónia Dias, Ana Rita Pedro, Ana Gama

**Affiliations:** 1 NOVA National School of Public Health, Public Health Research Centre (CISP), Comprehensive Health Research Center (CHRC), Associated Laboratory in Translation and Innovation Towards Global Health (REAL), Lisbon Academic Medical Centre (CCAL), NOVA University Lisbon, Lisbon, Portugal.

**Figure:**
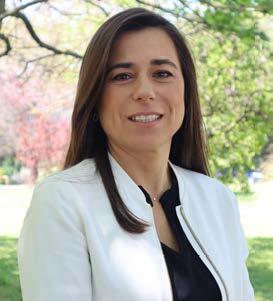

**Figure:**
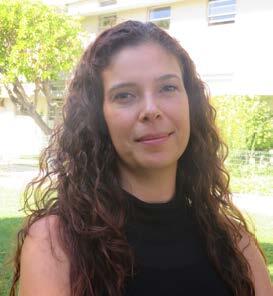

**Figure:**
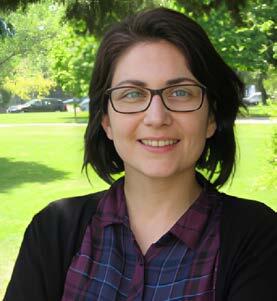

There is a strong body of evidence on the broad conditions that shape people’s health, the drivers that create, influence, or change those conditions, and the actions that can effectively improve populations’ health. Nevertheless, this knowledge remains mostly confined to publications within the science sphere, with limited real impact on population health indicators^1^. Indeed, the gap between scientific knowledge and policy and practice translates into delays between discover and adoption of evidence-based innovation that could improve quality of care, promote health and reduce inequalities. This is particularly relevant when health systems face challenges characterised by high complexity, such as population ageing, multimorbidity, health crises and persistent inequalities that press for results and require evidence-based effective solutions. In this context, Implementation Science (IS) has emerged as an interdisciplinary field seeking to answer fundamental questions such as: How can we ensure that what we know that works is effectively applied where it is needed? What do we know about the reasons behind the success of an intervention in a specific context? What explains that interventions that work in one setting fail in another? 

Implementation Science is defined as the scientific study of methods to promote the systematic adoption, integration and sustainment of evidence-based practices and interventions into routine practice in real-world settings^2^. IS offers tools to accelerate this transition, by combining the diagnosis of barriers and facilitators with the design and testing of strategies (through training, auditing and feedback, adaptation, co-creation, nudges, digital integration, governance). This involves understanding and addressing organisational, cultural, financial and behavioural barriers that hinder implementation^2^. IS contributes to effectiveness ensuring that interventions validated in controlled environments are adapted and remain effective when transferred to real world contexts. It improves efficiency and resources optimization by identifying cost-effective strategies for both the adoption of high-value practices and the de-implementation of low-value or obsolete ones. It advances equity by examining the barriers and promoting evidence-informed approaches to improve access, coverage, and adherence, which is particularly useful among vulnerable populations. Finally, it strengthens sustainability by supporting planning for long-term maintenance, integration into organisational workflows and information systems, and systematic monitoring of implementation outcomes such as fidelity, acceptability, and penetration^3^ ^-^ ^4^.

Over the past two decades, IS has generated a rich body of models and frameworks - ranging from determinant and process models to evaluation-oriented approaches - aimed at understanding and improving the systematic uptake, implementation, and sustainment of evidence-informed interventions in real-world settings^5^. Influential frameworks such as the Consolidated Framework for Implementation Research (CFIR), RE-AIM, Normalization Process Theory, and the Exploration, Preparation, Implementation, and Sustainment (EPIS) have moved the field beyond linear notions of knowledge translation, offering structured yet flexible lenses to examine implementation as a multilevel, dynamic, context-sensitive and non-linear process. Collectively, these frameworks offer analytically robust, yet pragmatically oriented tools for understanding how, why, and under what conditions interventions are embedded, routinized, and sustained in practice. Networks, centres, and training programmes that recognise IS as crucial for more resilient health systems are multiplying at an international level, highlighting the importance of the field. 

## Advances in Implementation Science in health in Portugal through collective action

The National School of Public Health of NOVA University Lisbon (NOVA NSPH), in Portugal, has contributing to a growing body of work in the field of IS in health. Recently, the Knowledge Centre in Implementation Science, based at NOVA NSPH, was created aimed at promoting evidence-to-practice integration, intersectoral collaboration, and capacity building. 

NOVA NSPH participates in multiple national and international funded research consortia and initiatives applying IS, that involve diverse health conditions (e.g. cancer, cardiovascular diseases and diabetes, HIV and other long term conditions), populations (e.g. experiencing heightened social vulnerabilities, with reduced health literacy, migrant background, youth and elderly) and settings (including primary/hospital healthcare units and community-based organizations within multisectoral ecosystems). Across these initiatives, IS approaches are employed to systematically map local needs and contextual determinants, such as organisational readiness, inequity drivers and structural barriers, that influence the adoption, implementation, success, scalability and sustainability of evidence-based interventions. By combining stakeholder-informed assessments with theoryinformed analyses, these efforts have generating actionable insights for improving disease prevention, early detection, and care pathways, particularly with a focus on underserved groups. This broad experience has demonstrating how IS can drive ethically grounded decisionmaking and support contextually attuned, responsive solutions, besides informing IS community internationally for contextdriven adaptation. 

The IS research carried out at NOVA NSPH also covers the co-design, adaptation, and evaluation of complex health interventions. By using mixed methods, participatory and co-creation frameworks, hybrid and pragmatic evaluation designs, researchers can collaboratively refine interventions and align them with local organisational processes, resources and end-user preferences. These approaches also facilitate a deeper understanding of implementation mechanisms, including fidelity, acceptability, feasibility, and contextual fit, and the interplay between intervention components and contextual influences, while enabling iterative optimisation during real-world delivery. Moreover, IS-driven work in digital health allows to address technological complexity, adoption barriers, and longterm viability within dynamic health systems, thus supporting the integration of digital tools into routine care. 

IS in Portugal has gained further momentum with the creation of the Portuguese Implementation Science Network, a platform under the Knowledge Centre. Recognising that the advancement of IS depends not only on rigorous empirical work but also on the dissemination of knowledge, the Network was created with the aim of gathering different stakeholders (e.g. users, professionals, managers, decision-makers, civil society organisations, industry and information technologies) to share evidence, co-learn, strengthen technical and cross-cutting skills, and foster innovation.

Building on this foundation, NOVA NSPH has expanded its work beyond IS research, provision of scientifictechnical support and dissemination, to include structured capacitybuilding initiatives to diverse stakeholders aimed at strengthening the institutional and workforce competencies across the broader health ecosystem. As such, the NOVA NSPH has developed a dedicated course on IS for multisectoral professionals focused on context assessment, cocreation, implementation strategies, and realworld evaluation, and has providing scientifictechnical advisory support to governmental agencies, health and social sector organisations, and community partners, thereby strengthening interdisciplinary learning and supporting the development of effective, equitable, and scalable health innovations. Overall, these complementary efforts contribute to expand implementation literacy and ensure that IS principles become embedded in policy, practice, and organisational decisionmaking.
