# Supplementary material for: Advances on Implementation Science in Health: Insights on trajectory, lessons learned and opportunities from the Portuguese experience
Source: Rev Lat Am Enfermagem. 2026 Jul 31;34:e5054. doi: 10.1590/1518-8345.0000.5054 (PMC13427258; doi:10.1590/1518-8345.0000.5054)
Supplement: Translation

# Translation: Avanços na Ciência da Implementação em Saúde: percepções sobre trajetória, lições aprendidas e oportunidades a partir da experiência portuguesa

**DOI:** 10.1590/1518-8345.0000.5055

**Authors:** Sónia Dias, Ana Rita Pedro, Ana Gama

**Affiliations:** 1 NOVA National School of Public Health, Public Health Research Centre (CISP), Comprehensive Health Research Center (CHRC), Associated Laboratory in Translation and Innovation Towards Global Health (REAL), Lisbon Academic Medical Centre (CCAL), NOVA University Lisbon, Lisbon, Portugal.

Existe um sólido corpo de evidências sobre as amplas condições que moldam a saúde das pessoas, os fatores que criam, influenciam ou modificam essas condições e as ações que podem melhorar efetivamente a saúde das populações. No entanto, esse conhecimento permanece, em grande parte, restrito às publicações no âmbito científico, com impacto real limitado nos indicadores de saúde populacional^1^. De fato, a lacuna entre o conhecimento científico e as políticas e práticas traduz-se em atrasos entre a descoberta e a adoção de inovações baseadas em evidências que poderiam melhorar a qualidade do cuidado, promover a saúde e reduzir desigualdades. Isso é particularmente relevante quando os sistemas de saúde enfrentam desafios caracterizados por elevada complexidade, como envelhecimento populacional, multimorbidade, crises em saúde e desigualdades persistentes, que pressionam por resultados e exigem soluções eficazes baseadas em evidências. Nesse contexto, a Ciência da Implementação (CI) emergiu como um campo interdisciplinar que busca responder a questões fundamentais, tais como: Como podemos garantir que aquilo que sabemos que funciona seja efetivamente aplicado onde é necessário? O que sabemos sobre as razões por trás do sucesso de uma intervenção em um contexto específico? O que explica que intervenções que funcionam em um cenário fracassem em outro?

A Ciência da Implementação é definida como o estudo científico de métodos para promover a adoção sistemática, a integração e a sustentabilidade de práticas e intervenções baseadas em evidências na prática rotineira em contextos reais^2^. A CI oferece ferramentas para acelerar essa transição, combinando o diagnóstico de barreiras e facilitadores com o desenho e o teste de estratégias (por meio de capacitação, auditoria e *feedback*, adaptação, coconstrução, *nudges* (incentivos sutis para influenciar comportamentos), integração digital e governança). Isso envolve compreender e enfrentar barreiras organizacionais, culturais, financeiras e comportamentais que dificultam a implementação^2^. A CI contribui para a efetividade ao assegurar que intervenções validadas em ambientes controlados sejam adaptadas e permaneçam eficazes quando transferidas para contextos reais. Ela melhora a eficiência e a otimização de recursos ao identificar estratégias custo-efetivas tanto para a adoção de práticas de alto valor quanto para a desimplementação de práticas obsoletas ou de baixo valor. Avança na equidade ao examinar barreiras e promover abordagens informadas por evidências para melhorar acesso, cobertura e adesão, o que é particularmente útil entre populações vulneráveis. Finalmente, fortalece a sustentabilidade ao apoiar o planejamento para manutenção de longo prazo, integração aos fluxos organizacionais e sistemas de informação, e monitoramento sistemático de desfechos de implementação, como fidelidade, aceitabilidade e penetração^3^ ^-^ ^4^.

Nas últimas duas décadas, a CI gerou um rico conjunto de modelos e *frameworks* - variando de modelos determinantes e processuais até abordagens orientadas para avaliação - voltados à compreensão e melhoria da incorporação sistemática, implementação e sustentabilidade de intervenções informadas por evidências em contextos reais^5^. *Frameworks* influentes, como o *Consolidated Framework for Implementation Research* (CFIR), RE-AIM, a *Normalization Process Theory* e o *Exploration, Preparation, Implementation, and Sustainment* (EPIS), moveram o campo para além das noções lineares de tradução do conhecimento, oferecendo lentes estruturadas, porém flexíveis, para examinar a implementação como um processo multinível, dinâmico, sensível ao contexto e não linear. Coletivamente, esses *frameworks* oferecem ferramentas analiticamente robustas, mas pragmaticamente orientadas, para compreender como, por que e sob quais condições as intervenções são incorporadas, rotinizadas e sustentadas na prática. Redes, centros e programas de formação que reconhecem a CI como crucial para sistemas de saúde mais resilientes multiplicam-se em nível internacional, destacando a importância do campo.

## Avanços da Ciência da Implementação em saúde em Portugal por meio da ação coletiva

A Escola Nacional de Saúde Pública da Universidade NOVA de Lisboa (NOVA NSPH), em Portugal, tem contribuído para um crescente corpo de trabalho no campo da CI em saúde. Recentemente, foi criado o Centro de Conhecimento em Ciência da Implementação, sediado na NOVA NSPH, com o objetivo de promover a integração entre evidência e prática, a colaboração intersetorial e o fortalecimento de capacidades.

A NOVA NSPH participa de múltiplos consórcios e iniciativas de pesquisa financiados em âmbito nacional e internacional que aplicam a CI, envolvendo diversas condições de saúde (por exemplo, câncer, doenças cardiovasculares e diabetes, HIV e outras condições de longa duração), populações (por exemplo, em situação de maior vulnerabilidade social, com baixa literacia em saúde, migrantes, jovens e idosos) e cenários (incluindo unidades de atenção primária/hospitalar e organizações comunitárias em ecossistemas multissetoriais). Ao longo dessas iniciativas, abordagens da CI são empregadas para mapear sistematicamente necessidades locais e determinantes contextuais, como prontidão organizacional, fatores geradores de iniquidades e barreiras estruturais, que influenciam a adoção, implementação, sucesso, escalabilidade e sustentabilidade de intervenções baseadas em evidências. Ao combinar avaliações informadas pelos *stakeholders* com análises orientadas por teoria, esses esforços têm gerado conhecimentos acionáveis para melhorar prevenção de doenças, detecção precoce e trajetórias de cuidado, particularmente com foco em grupos vulnerabilizados. Essa ampla experiência tem demonstrado como a CI pode impulsionar tomadas de decisão eticamente fundamentadas e apoiar soluções responsivas e ajustadas ao contexto, além de informar internacionalmente a comunidade da CI sobre adaptação orientada pelo contexto.

As pesquisas em CI realizadas na NOVA NSPH também abrangem o codesenho, adaptação e avaliação de intervenções complexas em saúde. Por meio do uso de métodos mistos, frameworks participativos e de coconstrução, desenhos híbridos e pragmáticos de avaliação, pesquisadores conseguem refinar colaborativamente intervenções e alinhá-las aos processos organizacionais locais, aos recursos disponíveis e às preferências dos usuários finais. Essas abordagens também facilitam uma compreensão mais profunda dos mecanismos de implementação, incluindo fidelidade, aceitabilidade, viabilidade e adequação contextual, bem como da interação entre componentes das intervenções e influências contextuais, ao mesmo tempo em que permitem otimização iterativa durante a implementação em cenários reais. Além disso, o trabalho orientado pela CI em saúde digital permite enfrentar a complexidade tecnológica, barreiras de adoção e viabilidade de longo prazo em sistemas de saúde dinâmicos, apoiando assim a integração de ferramentas digitais ao cuidado rotineiro.

A CI em Portugal ganhou ainda mais impulso com a criação da Rede Portuguesa de Ciência da Implementação, uma plataforma vinculada ao Centro de Conhecimento. Reconhecendo que o avanço da CI depende não apenas de trabalho empírico rigoroso, mas também da disseminação do conhecimento, a Rede foi criada com o objetivo de reunir diferentes *stakeholders* (por exemplo, usuários, profissionais, gestores, tomadores de decisão, organizações da sociedade civil, indústria e tecnologias da informação) para compartilhar evidências, aprender coletivamente, fortalecer competências técnicas e transversais e fomentar a inovação.

Com base nesse alicerce, a NOVA NSPH expandiu seu trabalho para além da pesquisa em CI, da prestação de suporte técnico-científico e da disseminação do conhecimento, passando a incluir iniciativas estruturadas de fortalecimento de capacidades voltadas a diversos *stakeholders*, com o objetivo de fortalecer competências institucionais e da força de trabalho em todo o ecossistema de saúde. Nesse sentido, a NOVA NSPH desenvolveu um curso específico em CI para profissionais multissetoriais, focado em avaliação de contexto, coconstrução, estratégias de implementação e avaliação em cenários reais, além de fornecer suporte técnico-científico consultivo a agências governamentais, organizações dos setores de saúde e social e parceiros comunitários, fortalecendo assim a aprendizagem interdisciplinar e apoiando o desenvolvimento de inovações em saúde efetivas, equitativas e escaláveis. Em conjunto, esses esforços complementares contribuem para expandir a literacia em implementação e garantir que os princípios da CI sejam incorporados às políticas, práticas e tomadas de decisão organizacionais.

# Translation: Avances en la Ciencia de la Implementación en Salud: perspectivas sobre la trayectoria, lecciones aprendidas y oportunidades desde la experiencia portuguesa

**DOI:** 10.1590/1518-8345.0000.5053

**Authors:** Sónia Dias, Ana Rita Pedro, Ana Gama

**Affiliations:** 1 NOVA National School of Public Health, Public Health Research Centre (CISP), Comprehensive Health Research Center (CHRC), Associated Laboratory in Translation and Innovation Towards Global Health (REAL), Lisbon Academic Medical Centre (CCAL), NOVA University Lisbon, Lisbon, Portugal.

Existe un sólido cuerpo de evidencia sobre las amplias condiciones que moldean la salud de las personas, los factores que crean, influyen o modifican dichas condiciones y las acciones que pueden mejorar efectivamente la salud de las poblaciones. Sin embargo, este conocimiento permanece en gran medida confinado a publicaciones dentro del ámbito científico, con un impacto real limitado sobre los indicadores de salud poblacional^1^. De hecho, la brecha entre el conocimiento científico y las políticas y prácticas se traduce en retrasos entre el descubrimiento y la adopción de innovaciones basadas en evidencia que podrían mejorar la calidad de la atención, promover la salud y reducir desigualdades. Esto es particularmente relevante cuando los sistemas de salud enfrentan desafíos caracterizados por alta complejidad, como el envejecimiento poblacional, la multimorbilidad, las crisis sanitarias y las desigualdades persistentes, que exigen resultados y requieren soluciones efectivas basadas en evidencia. En este contexto, la Ciencia de la Implementación (CI) ha emergido como un campo interdisciplinario que busca responder preguntas fundamentales tales como: ¿Cómo podemos garantizar que aquello que sabemos que funciona sea efectivamente aplicado donde se necesita? ¿Qué sabemos sobre las razones detrás del éxito de una intervención en un contexto específico? ¿Qué explica que intervenciones que funcionan en un entorno fracasen en otro?

La Ciencia de la Implementación se define como el estudio científico de métodos para promover la adopción sistemática, integración y sostenibilidad de prácticas e intervenciones basadas en evidencia en la práctica rutinaria en contextos reales^2^. La CI ofrece herramientas para acelerar esta transición, combinando el diagnóstico de barreras y facilitadores con el diseño y prueba de estrategias (mediante capacitación, auditoría y retroalimentación, adaptación, cocreación, *nudges* (empujones conductuales orientados a modificar comportamientos), integración digital y gobernanza). Esto implica comprender y abordar barreras organizacionales, culturales, financieras y conductuales que dificultan la implementación^2^. La CI contribuye a la efectividad asegurando que las intervenciones validadas en entornos controlados sean adaptadas y permanezcan efectivas cuando se transfieren a contextos reales. Mejora la eficiencia y la optimización de recursos al identificar estrategias costo-efectivas tanto para la adopción de prácticas de alto valor como para la desimplementación de prácticas obsoletas o de bajo valor. Avanza en la equidad al examinar barreras y promover enfoques informados por evidencia para mejorar el acceso, la cobertura y la adherencia, lo cual es particularmente útil entre poblaciones vulnerables. Finalmente, fortalece la sostenibilidad al apoyar la planificación para el mantenimiento a largo plazo, la integración en los flujos organizacionales y sistemas de información, y el monitoreo sistemático de resultados de implementación, como fidelidad, aceptabilidad y penetración^3^ ^-^ ^4^.

Durante las últimas dos décadas, la CI ha generado un rico conjunto de modelos y marcos conceptuales -que abarcan desde modelos determinantes y procesuales hasta enfoques orientados a la evaluación- destinados a comprender y mejorar la incorporación sistemática, implementación y sostenibilidad de intervenciones informadas por evidencia en contextos reales^5^. Marcos influyentes, como el *Consolidated Framework for Implementation Research* (CFIR), RE-AIM, la *Normalization Process Theory* y el *Exploration, Preparation, Implementation, and Sustainment* (EPIS), han llevado el campo más allá de las nociones lineales de traducción del conocimiento, ofreciendo lentes estructuradas pero flexibles para examinar la implementación como un proceso multinivel, dinámico, sensible al contexto y no lineal. Colectivamente, estos marcos ofrecen herramientas analíticamente robustas, pero pragmáticamente orientadas, para comprender cómo, por qué y bajo qué condiciones las intervenciones son incorporadas, rutinizaras y sostenidas en la práctica. Redes, centros y programas de formación que reconocen la CI como crucial para sistemas de salud más resilientes se multiplican a nivel internacional, destacando la importancia del campo.

## Avances de la Ciencia de la Implementación en salud en Portugal mediante la acción colectiva

La Escuela Nacional de Salud Pública de la Universidad NOVA de Lisboa (NOVA NSPH), en Portugal, ha contribuido a un creciente cuerpo de trabajo en el campo de la CI en salud. Recientemente, se creó el Centro de Conocimiento en Ciencia de la Implementación, con sede en la NOVA NSPH, con el objetivo de promover la integración entre evidencia y práctica, la colaboración intersectorial y el fortalecimiento de capacidades.

La NOVA NSPH participa en múltiples consorcios e iniciativas de investigación financiados a nivel nacional e internacional que aplican la CI, involucrando diversas condiciones de salud (por ejemplo, cáncer, enfermedades cardiovasculares y diabetes, VIH y otras condiciones de larga duración), poblaciones (por ejemplo, personas en situación de mayor vulnerabilidad social, con baja alfabetización en salud, migrantes, jóvenes y adultos mayores) y escenarios (incluyendo unidades de atención primaria/hospitalaria y organizaciones comunitarias dentro de ecosistemas multisectoriales). A través de estas iniciativas, los enfoques de CI son empleados para mapear sistemáticamente necesidades locales y determinantes contextuales, como preparación organizacional, factores generadores de inequidades y barreras estructurales, que influyen en la adopción, implementación, éxito, escalabilidad y sostenibilidad de intervenciones basadas en evidencia. Al combinar evaluaciones informadas por *stakeholders* con análisis guiados por teoría, estos esfuerzos han generado conocimientos accionables para mejorar la prevención de enfermedades, la detección precoz y las trayectorias de atención, particularmente con foco en grupos vulnerabilizados. Esta amplia experiencia ha demostrado cómo la CI puede impulsar decisiones éticamente fundamentadas y apoyar soluciones sensibles y adaptadas al contexto, además de informar internacionalmente a la comunidad de la CI sobre adaptación orientada por el contexto.

La investigación en CI desarrollada en la NOVA NSPH también abarca el codiseño, adaptación y evaluación de intervenciones complejas en salud. Mediante el uso de métodos mixtos, marcos participativos y de cocreación, diseños híbridos y pragmáticos de evaluación, los investigadores pueden refinar colaborativamente intervenciones y alinearlas con procesos organizacionales locales, recursos disponibles y preferencias de los usuarios finales. Estos enfoques también facilitan una comprensión más profunda de los mecanismos de implementación, incluyendo fidelidad, aceptabilidad, viabilidad y adecuación contextual, así como la interacción entre componentes de las intervenciones e influencias contextuales, permitiendo al mismo tiempo la optimización iterativa durante la implementación en escenarios reales. Además, el trabajo impulsado por la CI en salud digital permite abordar la complejidad tecnológica, las barreras de adopción y la viabilidad a largo plazo dentro de sistemas de salud dinámicos, apoyando así la integración de herramientas digitales en la atención rutinaria.

La CI en Portugal ganó aún más impulso con la creación de la Red Portuguesa de Ciencia de la Implementación, una plataforma vinculada al Centro de Conocimiento. Reconociendo que el avance de la CI depende no solo de un trabajo empírico riguroso, sino también de la difusión del conocimiento, la Red fue creada con el objetivo de reunir diferentes *stakeholders* (por ejemplo, usuarios, profesionales, gestores, tomadores de decisiones, organizaciones de la sociedad civil, industria y tecnologías de la información) para compartir evidencia, aprender colectivamente, fortalecer competencias técnicas y transversales y fomentar la innovación.

Sobre esta base, la NOVA NSPH ha ampliado su trabajo más allá de la investigación en CI, la provisión de soporte técnico-científico y la difusión del conocimiento, para incluir iniciativas estructuradas de fortalecimiento de capacidades dirigidas a diversos *stakeholders*, con el objetivo de fortalecer competencias institucionales y de la fuerza laboral en todo el ecosistema sanitario. En este sentido, la NOVA NSPH desarrolló un curso específico en CI para profesionales multisectoriales enfocado en evaluación de contexto, cocreación, estrategias de implementación y evaluación en escenarios reales, además de proporcionar apoyo técnico-científico consultivo a agencias gubernamentales, organizaciones de los sectores salud y social y socios comunitarios, fortaleciendo así el aprendizaje interdisciplinario y apoyando el desarrollo de innovaciones en salud efectivas, equitativas y escalables. En conjunto, estos esfuerzos complementarios contribuyen a expandir la alfabetización en implementación y garantizar que los principios de la CI sean incorporados en las políticas, prácticas y toma de decisiones organizacionales.
